# The complete mitochondrial genome sequence of the fairy ring-forming fungus *Lepista sordida*

**DOI:** 10.1080/23802359.2022.2067496

**Published:** 2022-04-25

**Authors:** Jae-Hoon Choi, Tomohiro Suzuki, Akiko Ono, Mihaya Kotajima, Yuki Tanaka, Toshiyuki Suzuki, Hirokazu Kawagishi, Hideo Dohra

**Affiliations:** a Research Institute of Green Science and Technology, Shizuoka University, Suruga-ku, Japan; b Graduate School of Science and Technology, Shizuoka University, Suruga-ku, Japan; c Graduate School of Integrated Science and Technology, Shizuoka University, Suruga-ku, Japan; d Center for Bioscience Research and Education, Utsunomiya University, Utsunomiya, Japan

**Keywords:** *Lepista sordida*, fairy ring-forming fungus, edible mushroom, basidiomycete, mitochondrial genome

## Abstract

*Lepista sordida* is a fairy ring-forming fungus that belongs to the family Tricholomataceae and is widely distributed in the Northern Hemisphere. Here, we report the complete mitochondrial genome sequence of *L. sordida*. The mitochondrial genome (57,375 bp) contained 20 protein-coding genes, 2 ribosomal RNA genes, and 26 transfer RNA genes. Phylogenetic analysis based on 14 conserved protein sequences from *L. sordida* and 15 related basidiomycetes showed that *L. sordida* was located on the outermost branch of the Tricholomataceae clade. This study is the first to report the complete mitochondrial genome sequence of a fairy ring-forming fungus belonging to the genus *Lepista.*

*Lepista sordida* (Schumach.) Singer 1951 is a mushroom belonging to the family Tricholomataceae (Kirk et al. 2008) and forms a circular pattern known as ‘fairy ring’ mainly on turfgrass, which is a phenomenon caused by the interaction between basidiomycete fungi and plants. To date, three compounds have been discovered and named as ‘fairy chemicals (FCs),’ which are involved in the formation of fairy rings (Choi et al. 2010a, 2010b, 2014; Kawagishi 2018). Genome analysis of *L. sordida* has revealed the biosynthetic pathway of FCs in this mushroom (Suzuki et al. 2016), and its genomic information is available on the web database F-RINGS (http://bioinf.mind.meiji.ac.jp/f-rings/) (Takano et al. 2019).

Mushrooms belonging to the genus *Lepista* are distributed worldwide and comprise approximately 50 species (Kirk et al. 2008); however, their limited morphological characteristics make it difficult to identify the species (Wang et al. 2019). In a previous study, the genus *Lepista* was divided into three clades by phylogenetic analysis based on multiple genes encoded in the nuclear genome (Alvarado et al. 2015). Comparative genome analysis and phylogenetic analysis of fairy ring-forming fungi, including *L. sordida*, will lead to a better understanding of the biological evolution of these fungi. However, it is not possible to confirm the results of phylogenetic analysis using the nuclear genome as there is no report of the complete mitochondrial sequence of the genus *Lepista*. In the present study, we determined the complete mitochondrial genome sequence of *L. sordida* and analyzed its phylogenetic relationship with related mushrooms.

*Lepista sordida*, which was originally collected from a lawn in Akita, Japan (40.2368 N, 140.5902 E), was deposited at Biological Resource Center (NBRC) of the National Institute of Technology and Evaluation (https://www.nite.go.jp/en/index.html, Culture Collection Division, nbrc-order@nite.go.jp) under voucher no. NBRC 112841. The mycelia of *L. sordida* were cultivated in potato dextrose broth with 0.5% yeast extract at 25 °C for 2 weeks, and genomic DNA was extracted using the cetyltrimethylammonium bromide (CTAB) method as previously described (Tanaka et al. 2017). A genomic DNA library was constructed using the TruSeq DNA PCR-Free Sample Preparation Kit, and 301 bp paired-end sequencing was performed using the MiSeq System (Illumina, San Diego, CA). Raw sequence reads were cleaned using Trimmomatic ver. 0.36 (Bolger et al. 2014) by trimming adapter sequences and low-quality ends (quality score, <15), and reads with a high *k*-mer coverage (>200) were extracted using Khmer ver. 2.0 (Crusoe et al., 2015). The resulting 677,450 read pairs totaling approximately 373.2 Mb were assembled using NOVOPlasty ver. 4.0 (Dierckxsens et al. 2017) with the nucleotide sequence of the cytochrome c oxidase subunit 1 gene from *Tricholoma flavovirens* (GenBank accession no. NC_046501.1 nt. 1–1584) as the seed sequence. The mitochondrial genome of *L. sordida* consisted of a circular DNA molecule of 57,375 bp in length with a GC content of 26.0%.

Gene prediction and annotation of the mitochondrial genome of *L. sordida* were performed using MFannot ver. 1.36 (http://megasun.bch.umontreal.ca/cgi-bin/mfannot/mfannotInterface.pl) and curated manually. A large subunit ribosomal RNA gene was predicted by alignment to the mitochondrial genome of *Coprinopsis cinerea* (NW_003307477.1, CC1G_23001) using Geneious Prime 2021 (Kearse et al. 2012). The mitochondrial genome of *L. sordida* contained 48 genes, including 20 protein-coding genes, 2 rRNA genes (*rnl* and *rns*), and 26 tRNA genes. Overall, 20 protein-coding genes encoded 14 conserved mitochondrial proteins (cox1–3, cob, nad1–6, nad4L, atp6, atp8, and atp9), ribosomal protein S3, and 5 hypothetical proteins, 2 of which showed similarities to RNA polymerase and LAGLIDADG homing endonuclease. Only a large subunit ribosomal RNA gene harbored an intron of 488 bp.

Phylogenetic analysis based on multilocus sequence analysis (MLSA) was performed using 14 conserved proteins (cox1–3, cob, nad1–6, nad4L, atp6, atp8, and atp9) from *L. sordida* and 15 related basidiomycetes (Figure 1). The amino acid sequences of 14 proteins were individually aligned using MAFFT v7.480 (Katoh and Standley 2013) with the –auto option. Next, poorly aligned regions were trimmed using trimAl v1.2 (Capella-Gutierrez et al. 2009) with the -automated1 option to improve subsequent phylogenetic analysis. The obtained alignments were used to generate a maximum-likelihood phylogenetic tree with IQ-TREE v1.6.12 (Nguyen et al. 2015) using 1,000 replicates with the ultrafast bootstrap approximation approach (UFBoot2) (Hoang et al. 2018) implemented in IQ-TREE. The phylogenetic tree showed that *L. sordida* was located on the outermost branch of the Tricholomataceae clade. In the future, it will be possible to further clarify the phylogenetic relationships of the genus *Lepista* by obtaining the genetic information of mushrooms belonging to this genus. This is the first report of the mitochondrial genome sequence of the genus *Lepista*, which will open new avenues for future studies on the evolution and classification of fairy ring-forming fungi.

**Figure 1.**
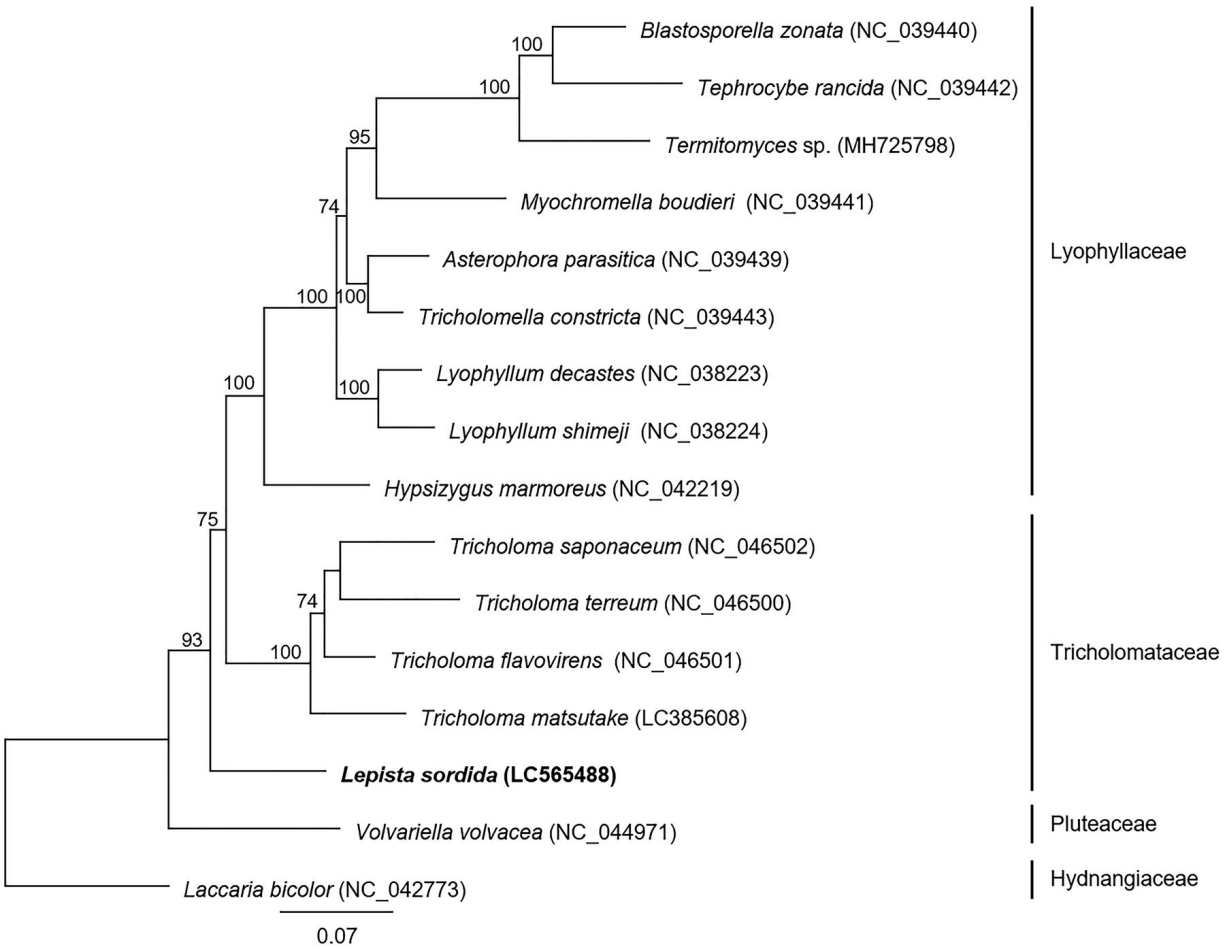
Molecular phylogenetic analysis of 16 basidiomycetes. The phylogenetic tree was constructed using the maximum-likelihood method with the amino acid sequences of 14 conserved mitochondrial proteins (cox1–3, cob, nad1–6, nad4L, atp6, atp8, and atp9). *Laccaria bicolor* was used as an outgroup species in the phylogenetic analysis. The accession numbers of the mitochondrial genome sequences used in this analysis are provided next to each species name. Bootstrap values higher than 70 are shown at the nodes. The scale bar indicates the number of substitutions per site.

## Data Availability

The genome sequence data that support the findings of this study are openly available in GenBank of NCBI (https://www.ncbi.nlm.nih.gov) under the accession number LC565488. The associated BioProject, SRA, and BioSample accession numbers are PRJDB4143, DRR327414, and SAMD00036984, respectively.
